# Fallibility, performance, patient safety and teamwork: embedding human factors in surgery

**DOI:** 10.1308/rcsann.2024.0007

**Published:** 2024-02-01

**Authors:** P Brennan, S Jarvis

**Affiliations:** 1 Consultant Maxillofacial Surgeon, Portsmouth Hospitals University NHS Trust, UK; 2 Specialist in Aviation Human Factors and Director of Jarvis Bagshaw Ltd, UK

Human fallibility occurs regularly – at home, at work and everywhere in between. We make errors all the time. Learning and sharing lessons from medical error is important to avoid them in future or mitigate the potential harm. We would encourage reporting mistakes to the Confidential Reporting System in Surgery (CORESS). This can be easily done online via coress.org.uk. Nevertheless, we can never eliminate error completely, which is why in our view, the term ‘never event’ (defined by the NHS as a serious incident that is wholly preventable because guidance or strong systemic protective barriers are available and should have been implemented)^1^ is a misnomer.

In healthcare, approximately 1 in 20 hospital admissions has some form of error.^2^ This might be something relatively minor, like forgetting to arrange an outpatient follow-up appointment or complete a discharge summary. Serious medical or surgical errors occur in approximately 1 in 400 admissions.^2^ Many of these might be prevented by understanding and applying human factors (HF) in our surgical practice.

## What are the types of error in surgery?

Most errors are multifactorial, originating long before they actually occur. For example, organisational issues (so called latent failures) such as overbooked clinics or operating lists could result in corners being cut, something being missed or miscommunication and result in a later error.

At an individual or surgical team level, errors and mistakes are common. Skill-based errors happen during familiar processes (e.g. using the wrong diathermy foot pedal, or retaining a swab or instrument). While it is tempting to attribute this to complacency or inattention, all of us are vulnerable to these errors in distracting, multitask environments because our attention cannot be on everything at once. Mistakes (categorised as ‘rule-based’ or ‘knowledge-based’) are where decisions or judgements go astray. For example, a rule-based mistake could occur by prescribing a standard drug dose but misjudging the patient’s weight. A knowledge-based mistake might be a wrong diagnosis, followed by an incorrect procedure.

In addition to errors and mistakes, violations are very common in all safety critical industries. Surprisingly, almost all are well intended and based on experience or are essential workarounds. However, where the bases of rules and practices are not well understood or where known risk is accepted, violations can cause serious harm. Examples include continuing to operate knowing there is a hole in a glove, not wearing appropriate equipment or not engaging with checklists. Fortunately, only a very small minority of professionals go to work with the intent of causing deliberate patient harm. Ian Paterson is a well-known example in surgery.

## So what are HF and why are they so important to surgeons?

HF is an umbrella term for many different interpretations and definitions. For now, we will consider HF as how humans work, how we interact with others, and how our analysis, decision making and behaviour can potentially adversely affect patient care, safety and professional relationships. Good systems and processes (including checklists, protocols, fail safes and other measures) are necessary to help reduce error.

HF includes systems design, ergonomics (how we interact with equipment and technology including robots) and human performance. HF experts may specialise in one or more areas, such as designing complex systems or the psychology of human performance. Most surgeons will not be formally trained in HF design but we can apply the many factors that affect individual and team performance to improve patient safety and teamworking.

The General Medical Council (GMC) recognises the importance of HF education and training, and include them in the generic professional capabilities needed for safe medical practice.^3^ The GMC also acknowledges HF as potentially contributory causes in fitness to practise referrals.^4^

## What are some of the HF relevant to surgery?

Some of the most important areas that affect individual and surgical team performance include effective and unambiguous communication, maintaining situation awareness (what is happening around us), managing our workload, recognising the effects of distraction and looking at how our performance deteriorates slowly over time.

### Communication

Many mistakes occur because of poor communication. This is a major contributor to NHS never events, including wrong site surgery and retained swabs.^5^ As the ‘sender’ of information, we might assume that it has been heard (or read) and understood by the ‘receiver’ but this might not be the case. Ambiguity can occur in both written documentation and verbal instructions that are misinterpreted or even not heard because of background noise or distraction. We recommend avoiding pronouns (such as it, that, these, those) during safety critical times. It is much better to use proper nouns when asking for instruments, equipment, implants or drugs and when giving specific instructions. ‘Repeat back’ or interpretation by the receiver is a useful way to ensure instructions are heard and understood. If there is any doubt, the safest option is to briefly stop what we are doing and seek clarification.

### How can we optimise our performance?

Optimising performance can improve patient safety and reduce surgical errors. Colleagues who do not eat breakfast are essentially in a fasting state, burning body fat and generating ketones. Similarly, missing lunch can result in the same biochemical fasting state and it is well known that this can reduce performance.^6^ A 1–2 kg loss in total body water due to lack of rehydration can reduce analysis and decision making by up to 20%.^7^ Taking short breaks every 3–4 hours during a long, complex operation to eat, drink and regain energy can help to maintain performance levels. Most would not drive for more than a few hours before stopping and yet it seems to be acceptable to operate for very long periods. Have you asked your team how they feel about operating for 6, 7 or even 8 hours non-stop?

When something does not seem quite right (and if safe to do so), stopping, stepping back and reappraising the situation before continuing can reduce the chance of error. It might sound obvious when not actually in a stressful environment. However, when performing under pressure, this simplest of actions can be overlooked. An easily remembered mini brief called PPP (patient, procedure, people) can be useful to focus team discussion.^8^

### Behaviour towards others

Incivility, bullying, sexual misconduct and discrimination are not HF in its purest definition but these behaviours affect performance, decision making and team morale, and they can have hugely detrimental effects.^9,10^ Similarly, shouting and venting anger, particularly during stressful times, increases the risk of error, not to mention its effect on the team. Most subsequently regret these outbursts. The evolutionary primitive limbic system can ‘hijack’ higher brain functions in such circumstances. It is much better to stop, think and let higher functions catch up before acting. In this way, we maintain the respect of the team. Likewise, lowering authority gradients and actively empowering staff so that anyone can question or challenge more senior colleagues without fear of retribution is good practice and can also improve patient safety.^11^

### Avoiding error traps

Many other factors including time constraints, disruption to circadian rhythms during night shifts, fatigue and tiredness, confirmation bias, distraction and multitasking can all raise the risk of error. We recommend that colleagues are mindful of the signs of fatigue and burnout as well as looking out for each other.^12^ An interesting study on burnout in trauma and orthopaedic surgeons is published in this issue of the *Annals*.^13^

Confirmation bias is where we naturally use information (e.g. diagnostic tests or relevant surgical anatomy) to confirm our decisions or actions, rather than challenging or reflecting on them. This is another example of where good teamworking, and valuing trainee and other staff input and suggestions can help avoid potential problems or errors.

**Figure 1:**
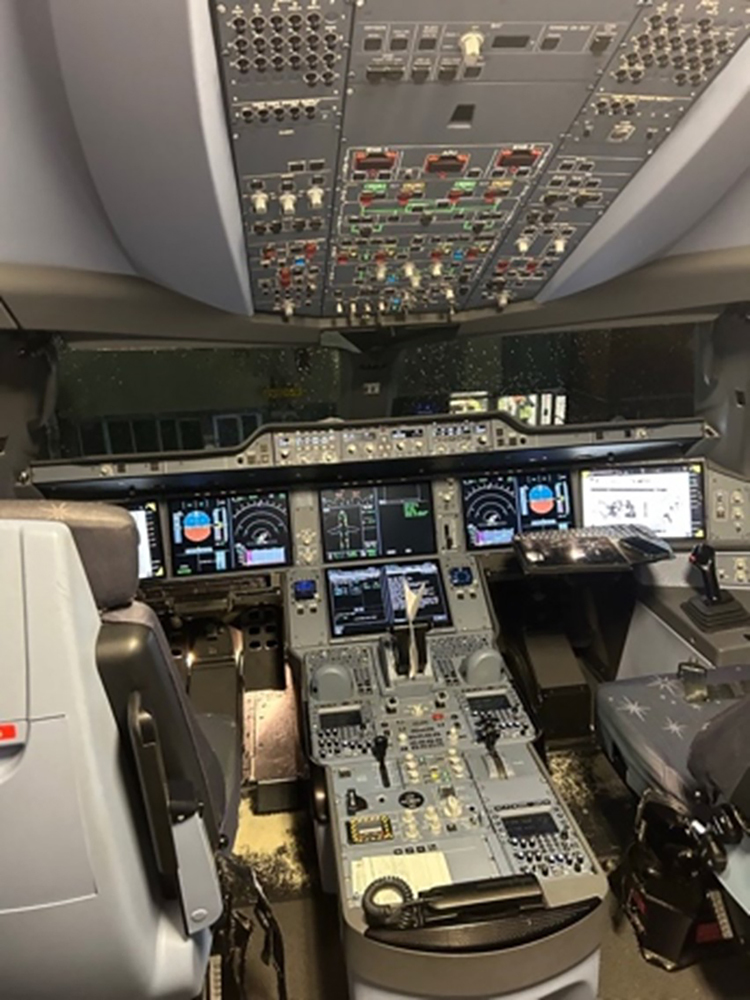
On the flight deck (in this case, an Airbus A350), the crew is not interrupted or distracted during safety critical times. Distraction should also be minimised where possible during complex surgery.

Distraction occurs frequently in surgery and can be a significant cause of error. A study published in 2020 found that fewer than half of invasive cardiac catheter procedures were completed without distraction and many were during high-risk stages of procedures.^14^ When intense concentration is required during surgery, minimal or no distraction is recommended. This practice is used routinely by other high-reliability organisations during critical times and has resulted in significant improvements to safety (Figure 1).

**Figure:**
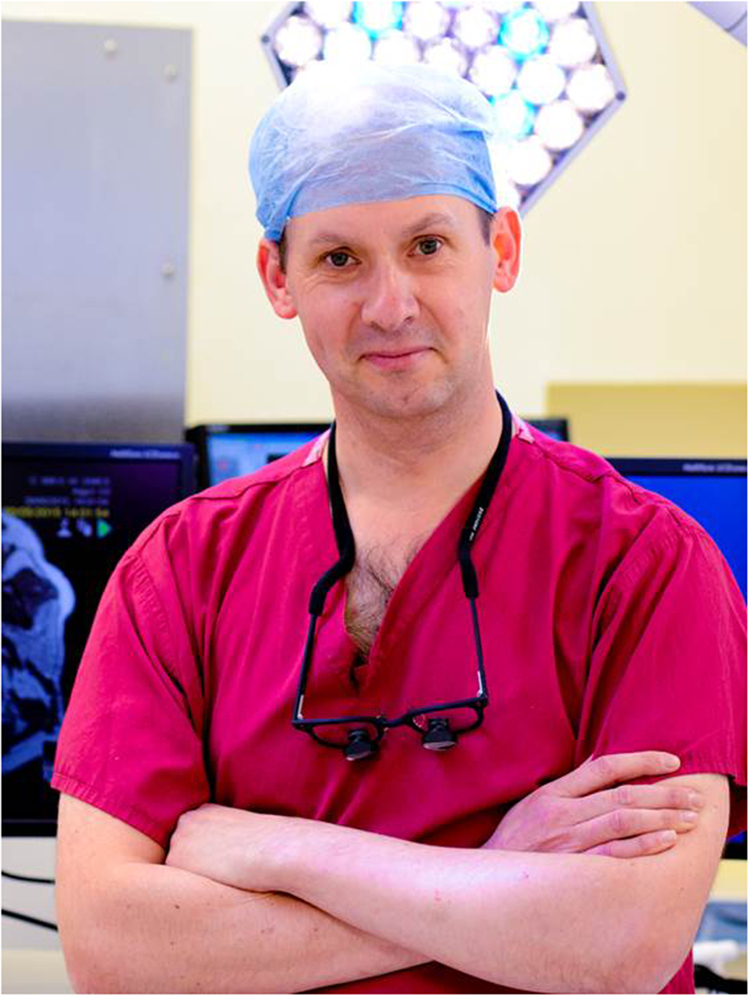
**P Brennan**, Consultant Maxillofacial Surgeon, Portsmouth Hospitals University NHS Trust, UK.

**Figure:**
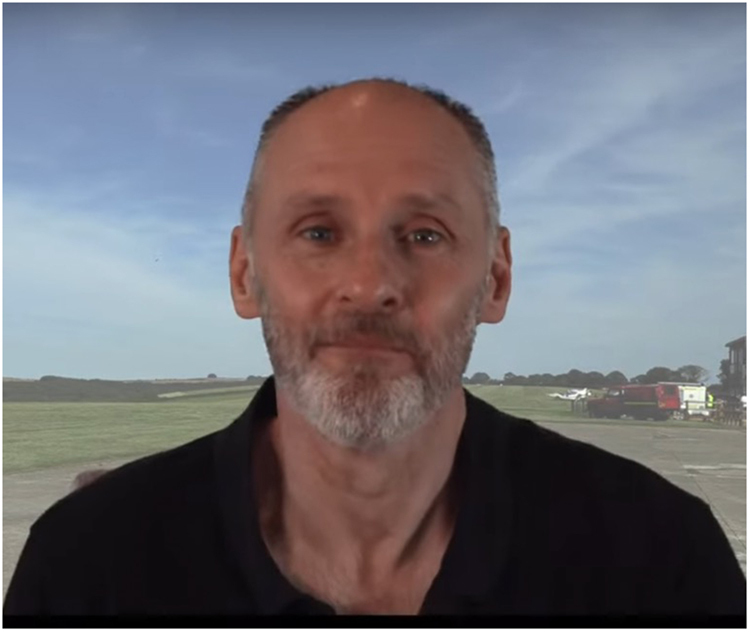
**S Jarvis**, Specialist in Aviation Human Factors and Director of Jarvis Bagshaw Ltd, UK.

## The value of the team briefing

This is a great opportunity to build good working relationships, create a ‘safe space’ for staff and actively engage with checklists as if we were on the operating table. It is good practice to think about ‘what if?’ scenarios to avoid potential startle reactions and this can help build situation awareness. Being aware of what is going around us is a dynamic process that can deteriorate for both individuals and teams, with tragic consequences.

As with other high-reliability organisations, healthcare needs a ‘just culture’ so that when problems arise, they can be discussed in an open and non-accusatory manner. The approach to learn from mistakes should be ‘why did this happen?’ rather than ‘who was to blame?’. In this regard, acknowledging fallibility and understanding HF in daily practice is so important to help reduce preventable error as well as enhancing patient safety, teamworking and morale.

Awareness of HF and the benefits of applying them in surgery is gaining increasing traction. Over many years, most surgeons have focused their research on the many technical aspects of surgery, improving the understanding of disease processes and advances in patient care. However, the value and importance of HF and non-technical skills research in surgery cannot be underestimated. Poor communication is one of the most common reasons for significant surgical error, and further research is needed to better understand how and why it happens so frequently. More studies are also required on team dynamics and interaction including during robotic surgery. As has been found recently, distraction can be a potential cause of significant error in the cardiac catheter laboratory^14^ and this work could be replicated in different operating theatres.

There are so many other HF areas that can be researched around analysis and decision making, how we process information and the effect of biases on surgical performance. With fatigue becoming a well-recognised issue across healthcare, further work in this area for surgical teams would also be invaluable.

We hope that colleagues will not only recognise the value of HF in their practice but also contribute to advancing knowledge and understanding in this ever growing and fascinating science. The *Annals* is definitely keen to publish more HF papers in future!
